# Modification of Light-Cured Composition for Permanent Dental Fillings; Mass Stability of New Composites Containing Quinoline and Quinoxaline Derivatives in Solutions Simulating the Oral Cavity Environment

**DOI:** 10.3390/ma17236003

**Published:** 2024-12-07

**Authors:** Ilona Pyszka, Beata Jędrzejewska

**Affiliations:** Faculty of Chemical Technology and Engineering, Bydgoszcz University of Science and Technology, 85-326 Bydgoszcz, Poland

**Keywords:** dental material, light-curing composition, photopolymerization, mass stability, sorption, solubility

## Abstract

Billions of patients struggle with dental diseases every year. These mainly comprise caries and related diseases. This results in an extremely high demand for innovative, polymer composite filling materials that meet a number of dental requirements. The aim of the study was to modify the light-cured composition of permanent dental fillings by changing the composition of the liquid organic matrix. New photoinitiators (DQ1-DQ5) based on a quinoline or quinoxaline skeleton and a co-initiator-(phenylthio)acetic acid (PhTAA) were used. In addition, monomers that have been traditionally used in dental materials were replaced by trimethylolpropane triacrylate (TMPTA). The neutral dental glass IDG functioned as an inorganic filler. The influence of the storage conditions of the developed composites in solutions simulating the natural oral environment during the consumption of different meals on sorption, solubility, and mass changes was assessed. For the tests, fifty-four cylindrical composite samples were prepared according to ISO 4049 guidelines and stored in different solutions. Distilled water, artificial saliva, heptane, 10% ethanol, and 3% acetic acid, as well as solutions containing pigments such as coffee, tea, red wine, and Coca-Cola, were used for the studies. The samples were stored in these solutions for 7, 14, 28, 35, 42, 49, 56, and 63 days at 37 °C. The sorption, solubility, and mass changes in the tested samples were determined, and the trend of these changes as a function of storage time was presented. The results were analyzed considering the nature of the solution used, i.e., aqueous, hydrophobic, and acidic. The properties evaluated changed in a different way, characteristic for each of the abovementioned solution groups. It was found that the type of solution simulating the natural environment of the oral cavity has the greatest influence on the sorption, solubility, and changes in the mass of the tested material.

## 1. Introduction

Dental caries is the most common oral disease in the world, affecting both adults and children. According to statistics presented on the most important dental event, World Oral Health Day (WOHD), it is estimated that despite treatment and continuous improvement in prevention, as many as 2.4 billion people have caries of permanent teeth, and 530 million children suffer from caries of deciduous teeth [1,2]. In conservative dentistry, the treatment of tooth decay involves removing the infected dentin and restoring the structure and function of the tooth using an appropriate material. Choosing the right materials for dental fillings is crucial to the success of dental treatment. Dental fillings, necessary to rebuild tooth structure after caries removal, must meet a number of requirements, including strength, aesthetics, and compatibility with tooth tissues, etc. [3,4,5,6]. The materials available on the market differ in their properties and applications, which allows the solution to be individually tailored to the patient’s needs. The growing demand for the aesthetic reconstruction of patients’ teeth has resulted in a rapid increase in the use of polymer composite materials for fillings [7].

Classic composite dental fillings consist of two main components. It is a liquid organic matrix that undergoes cross-linking polymerization and a powdered inorganic filler influencing the final properties of the composite.

The liquid organic matrix consists of a photoinitiator, co-initiator, solvent, and a multifunctional monomer [8,9,10,11,12,13,14,15], which guarantee a high degree of cross-linking. The most-used monomers in dental compositions currently available on the market (RCB—resin-based composites) include monomers such as bisphenol A diglycide ether dimethacrylate (Bis-GMA) and triethylene glycol dimethacrylate (TEGDMA). Bis-GMA is used in dental materials due to the presence of the aromatic structure of Bisphenol A in the core of the molecule, which ensures low volatility and high modulus of the hardened composite. TEGDMA, on the other hand, acts as an active diluent due to its low viscosity, which allows the introduction of an appropriate amount of inorganic filler. The weight ratios of both monomers in the composition are most often 7:3 or 8:2, where Bis-GMA is the main component. The application of co-initiators (electron donors) in the presence of photoinitiators (electron acceptors) that generate free radicals [16] in the photoinduced intermolecular electron transfer (PET) process [17,18,19] is necessary due to the way that composite materials are hardened. Currently, the most common method is photopolymerization, in which light initiates the chain reaction [9,11]. The most commercially used photoinitiator is camphorquinone (CQ), which is sensitive to the blue light emitted by dental lamps [9,11,14,16]. However, its drawback is the intense yellow color due to the absorption band in the range of 460–480 nm [20,21]. Moreover, in order to increase its reactivity, the presence of a co-initiator in the liquid organic matrix is necessary [9,11,16]. In commercial products, tertiary amines are used as co-initiators, mainly 4-N,N-dimethylaminophenylethanol or ethyl 4-dimethylaminobenzoate [9,14].

The second main component of dental composite fillings are inorganic fillers. Most often, these are various types of dental glass characterized by excellent transparency and quartz.

In addition, dental fillings contain inhibitors to prevent premature polymerization during storage, photostabilizers to stop changes in the color, and compounds that allow the color of the filling to be matched to the natural color of the patient’s teeth [8,9,10,14,15].

Modern dentistry places enormous demands on composites with potential dental applications. Due to the many inconveniences in using commercial polymer composites, the need arose to develop new, universal, and effective materials. These inconveniences result primarily from the widespread use of the Bis-GMA monomer. Many studies have shown that approximately 25–50% of unsaturated methacrylate groups remain unpolymerized in composites. The unreacted monomer can escape from the organic matrix and diffuse into surrounding tissues [22,23]. Furthermore, even small doses of Bisphenol A have a negative impact on the human body. It has been proven that its presence in the body disturbs the natural hormonal balance and causes problems with obesity, psychomotor hyperactivity, reduced fertility, miscarriages, and immune disorders [24,25,26,27,28]. Moreover, Bis-GMA can penetrate various types of disposable gloves used by medical personnel, causing allergic reactions with changes on the fingertips such as epidermal exfoliation and deep cracks [29].

Another aspect worth paying attention to is the presence of tertiary aromatic amines in commercial products (i.e., ethyl 4-dimethylaminobenzoate-EDMAB), which are often geno- and cytotoxic factors [30]. Moreover, according to the literature [31,32], the high concentration of camphorquinone in composite materials affects the aesthetics of the filling and the quality of the final product, e.g., causing the yellow color of the resulting filling.

Polymer restorative materials are overly complex compositions that contain a number of reagents with specific functions. Therefore, in the constantly changing environment of the oral cavity, unreacted components of dental fillings may be washed out [33,34]. In addition, the use of certain oral hygiene products, e.g., bleaches, also contributes to the destruction of the structure of fillings and the release of compounds contained in them [35]. Before any designed polymer material is intended to come into contact with the human body, it must be thoroughly examined, and the potential negative health effects must be eliminated.

The oral cavity, which is the target place for the use of dental fillings, is an extremely diverse environment. Therefore, the properties of polymeric restorative materials may also depend on many factors occurring in their environment. Mass stability is a key factor determining the durability of the filling used.

Since the oral cavity, which is the target site for dental fillings, is an extremely diverse environment, the properties of polymeric restorative materials can depend on many factors related to it. Mass stability is a key factor in determining the durability of the filling used.

The influence of the oral environment on the mass stability of polymer dental fillings depends on the hydrophilicity of the polymer matrix. As it increases, the material’s tendency to sorb water and aqueous solutions from the environment increases [36,37,38,39,40,41]. Hydrophilic matrix components have the ability to form hydrogen and polar bonds, causing water to be retained in the material structure [36,38]. This parameter also depends on the porosity and density of the polymer network. It is related to the penetration of solvent molecules through the pores of the material and migration to accessible places. Most often, these are large intermolecular spaces of polymers with lower cross-linking density. In practice, this phenomenon can be minimized by increasing the content of inorganic filler, since the sorption and solubility are mainly attributed to the polymeric, organic part of the composite [36].

In addition to material factors, the type of solution in contact with the filler affects the mass stability. It has been observed that the solubility is greater in the presence of acidic and alcoholic solutions than in water [36,37]. Furthermore, acidic solutions often cause erosion of the filler surface, resulting in a reduction in the composite mass [38]. On the other hand, aqueous solutions with different characteristics are most common. Through diffusion, they can penetrate the structure of polymeric materials for restoration, initiating chemical and physical processes. As a result, they lead to changes in their volume, swelling, plasticization, softening, oxidation, hydrolysis, etc. [36].

The presented work is a continuation of research conducted in the field of developing new, innovative dental fillings. In our previous paper [42], we described high-performance polymer composites obtained by changing the components of the polymer matrix, which includes new initiators and co-initiators as well as monomers. Since currently used commercial materials for the reconstruction of hard tooth tissues are not ideal, novel solutions are sought to meet the requirements of dentistry. The emphasis is on modifications of the chemical composition of polymeric materials, both in their matrix and filler. The aim of this work was to develop new polymer composites and then to investigate the mass stability of the hard fillings obtained. In the first part of the article, we described composites obtained based on new photoinitiating systems without inorganic filler. Their ability to photoinitiate polymerization was compared with a model system containing commercial components. As photoinitiators, we used newly designed organic compounds based on quinoxaline and a quinoxaline skeleton. They have nitrogen-based heterocyclic rings, which can exert biological, chemotherapeutic and pharmacological activities [43,44] and show anticancer, antibacterial, antiviral, antimalarial, and antifungal properties [45,46,47,48,49]. Instead of aromatic amines, we applied (phenylthio)acetic acid (PhTAA) as a co-initiator. In addition, we used trimethylolpropane triacrylate (TMPTA) as a monomer, replacing the monomers traditionally used in dental composites—Bis-GMA, UDMA (urethane-dimethacrylate), TEGDMA (triethylene glycol dimethacrylate), HEMA (hydroxyethylmethacrylate). Next, we prepared fifty-four cylindrical polymerized composite samples that stimulated fillings of hard dental tissues. They contained, in addition to an organic matrix, an inorganic filler. We then determined the mass stability of the obtained materials in solutions simulating the natural oral environment, such as distilled water, artificial saliva, coffee, tea, red wine, and Coca-Cola.

## 2. Materials and Methods

### 2.1. Reagents

The synthesis of photoinitiators DQ1-DQ5 used in the studies was described in our previous paper [42]. The monomers used, namely trimethylolpropane triacrylate (TMPTA), bisphenol A glycerolate dimethacrylate (Bis-GMA); the commercial photoinitiator, namely camphorquinone (CQ), and the electron donors (co-initiators), namely (phenylthio)acetic acid (PhTAA), ethyl 4-dimethylaminobenzoate (EDMAB), were all purchased from Sigma-Aldrich Co. (St. Louis, MO, USA). The solvents, i.e., acetic acid, ethanol, methanol, heptane, 1-methyl-2-pyrrolidinone (MP), were supplied by Merck. Salivia sintetica CTS was purchased from C.T.S. s.r.l. Company, Rovigo, Italy.

The structures of the compounds used for photoinitiated polymerization studies are presented in Table 1.

Neutral dental glass was used as an inorganic filler in polymer composites-IDG (Inter Dental Glass) GM 35429 from Schott Dental Glass Co., Wolverhampton, UK, with composition (in % by mass): SiO_2_—30, CaO—10, Al_2_O_3_—30, F—15, P_2_O_5_ < 10, Na_2_O < 10. The choice of this glass filler was due to its high quality, purity, and excellent transparency.

### 2.2. Methods

#### 2.2.1. Preparation of Samples for Testing

Two types of polymerizing mixtures were prepared, one for polymerization kinetics studies, and the other for mass stability tests. The quantities of individual components for the preparation of photocurable compositions, both developed and commercial, are listed in Table 2.

The photocurable mixture for polymerization kinetics studies contained 0.1 mL of 1-methyl-2-pyrrolidone (MP), which acted as a solvent, and 0.9 g of trimethylolpropane triacrylate (TMPTA) monomer. The photoinitiators (electron acceptors) in the photoinitiating systems studied were DQ1-DQ5 compounds, the synthesis of which was described in our previous paper [42]. Depending on the molar absorption coefficient, their concentration ranges from 1.35 × 10^−3^ to 1.80 × 10^−3^ M. An acetic acid derivative—PhTAA at a concentration of 0.1 M was used as a co-initiator. The effectiveness of initiating the polymerization by the tested systems was compared with samples containing the commercial photoinitiator camphorquinone (CQ) and the co-initiator ethyl 4-dimethylaminobenzoate (EDMAB), commonly used in dentistry. The concentration of camphorquinone was 0.675 M, while the concentration of the co-initiator was 0.1 M.

The materials prepared for mass stability tests corresponded to commercial dental fillings. Therefore, in addition to the organic matrix, the photocurable compositions contained an inorganic filler. Neutral dental glass (IDG) was used as the filler, which was added to each photoinitiating composition in the amount of 1.50 g.

#### 2.2.2. Polymerization Kinetics Studies

The photopolymerization process was conducted in a specially prepared Teflon ring with a diameter of 10 mm and a thickness of 3 mm (Teflon ring area 0.785 cm^2^). To prevent the sample from flowing out, a glass plate (microscope cover slip, Chem-Land) was glued to the Teflon ring on one side using silicone grease (Bayer Baysilone Paste, Bayer, Leverkusen, Germany). Then, the prepared photocurable composition was placed in the ring and irradiated from below with blue light (390–500 nm) emitted by a dental LED polymerization lamp (Cromalux 75 Mega Physik Dental, Rastatt, Germany). The power of the light beam was measured at 400 nm using a FieldMaster Coherent Laser Power and Energy Meter and was 20 mW/cm^2^. The distance of the sample from the light source was the same for all measurements.

A microcalorimetric method was used to study the kinetics of TMPTA photopolymerization as described in our previous papers [42,50]. In these tests, the photocurable compositions contain only an organic matrix, without filler. A thermocouple (RTD Thermometer Delta OHM HD 2107.1, Atlanta, GA, USA) was inserted into the polymerizing sample placed in a Teflon ring from the top so that its tip was in contact with the surface of a glass plate. The time needed to stabilize the temperature in the sample before irradiation was 10 s and was the same for all samples. The temperature change was recorded every 1 s for 40 s. The recorder (Delta OHM HD 40.1, Atlanta, GA, USA) enabled temperature measurement with an accuracy of ±0.1 °C. Three measurements were made for each of the tested materials. The initial polymerization rates were determined as a slope of the linear part of the kinetic curve (temperature versus time curve) at its initial time to avoid the possibility of non-isothermal reaction conditions [42,50]. The obtained values were then converted to data in μmol/s.

#### 2.2.3. Methodology for Testing Mass Stability

For mass stability tests, fifty-four cylindrical polymerized composite samples containing an organic matrix and an inorganic filler were prepared in a similar manner as described above using a Teflon ring as a mold. The samples, being equivalents of dental fillings, were placed in solutions simulating the natural oral environment during consumption of various meals, the characteristics of which are given in Table 3. Such tests are most often performed in distilled water [51,52,53] and artificial saliva [54,55], but also in solutions containing pigments such as coffee, tea, red wine, or Coca-Cola [52,53,55,56].

The tea solution was prepared by pouring 1 bag of black express tea (Lipton Earl Gray, CVC Capital, St Helier, Jersey) with 250 mL of boiling water and infusing for 3 min (according to the manufacturer’s instructions). The coffee solution was prepared by pouring 2.5 g of instant coffee (Jacobs Kronung, Jacobs Douwe Egberts, Amsterdam, Netherlands) with 250 mL of freshly boiled hot water (according to the manufacturer’s instructions). The red wine used was Porta da Ravessa, Adega Cooperativa de Redondo, Redondo-Évora, Portugal.

In accordance with the PN-EN ISO 4049 standard, which defines the method of determination and the limit values of sorption and solubility of polymeric restoration materials [57], all samples were weighed (*m*_1_). Then, the prepared samples were placed in 10 mL of the solutions listed in Table 3 and conditioned at 37 °C for 7, 14, 21, 28, 35, 42, 56 and 63 days. After the specified storage time, the samples were dried using a paper towel to remove the solution absorbed on the surface, weighed (*m*_2_), and then dried in a desiccator to constant weight (±0.1 mg) at 37 °C in the presence of a drying agent, namely silica gel. After reaching a constant mass, the samples were weighed again (*m*_3_). After each completed cycle, the samples were placed back in freshly prepared solutions. The time after which subsequent values of sorption, solubility, and mass changes were determined was summed up.

Sorption (*S_p_*) was calculated based on Equation (1) as follows:

(1) $$S_{p}=\left(\frac{m_{2}-m_{3}}{m_{2}}\right)\cdot 100\%$$

where

*S_p_* is the mass that is reversibly adsorbed during storage in relation to the mass of the swollen sample.

Solubility (*S_l_*) was calculated using Equation (2):

(2) $$S_{l}=\left(\frac{m_{1}-m_{3}}{m_{1}}\right)\cdot 100\%$$

where

*S_l_* is an irreversible change in mass, unchanged after drying in a desiccator in relation to the initial mass of the sample.

Mass changes were calculated using Equation (3) as follows:

(3) $$D_{m}=\frac{m_{2}-m_{1}}{m_{1}}\cdot 100\%$$

where

*D_m_* is the change in mass during storage before drying in relation to the initial mass of the sample.

As indicated by many authors [37,38,39,40,58,59,60,61], full saturation of dental polymer composites usually occurs after a longer period of time; exceeding this does not cause significant changes in the values of both parameters, i.e., sorption and solubility. Therefore, the tests were conducted with an incubation time extended to 63 days compared to that recommended by the standard. Moreover, many authors use certain simplifications in calculations, based only on the mass of the samples (ignoring their volume), which allows for a clearer presentation of the percentage of sorption and solubility values [37,40,58,60,62].

## 3. Results and Discussion

### 3.1. Photopolymerization

Despite their many advantages, the currently used commercial compounds are not perfect composite materials. Therefore, modern dentistry is constantly looking for new compounds that can replace them. The modification of the properties of the composites we proposed was achieved by changing the liquid organic matrix. It involved the use of new photoinitiators and a co-initiator. The photoinitiators were organic compounds based on a quinoline or quinoxaline skeleton (DQ1–DQ5). Moreover, the aromatic amines currently used as co-initiators were replaced by (phenylthio)acetic acid (PhTAA), and trimethylolpropane triacrylate (TMPTA) was applied as the monomer instead of Bis-GMA.

**Figure 1:**
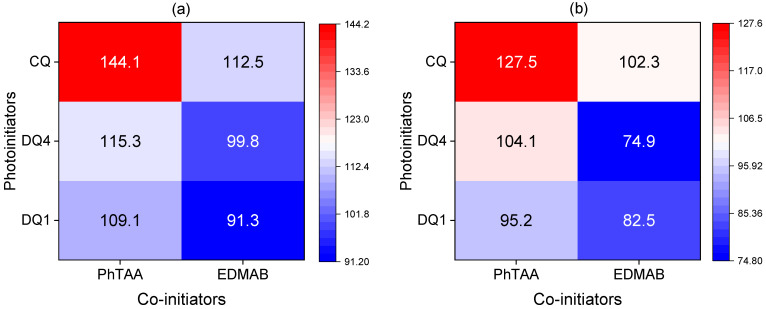
Heat maps of polymerization rates (μmol s^−1^) obtained during photoinitiated polymerization using initiators: DQ1, DQ4 and CQ, co-initiators: PhTAA and EDMAB (0.1 M) and monomers: (**a**) TMPTA, (**b**) Bis-GMA. The light po of the dental lamp was 20 mW cm^−2^; the right panel of the graphs shows the initial polymerization rate gradient; the numbers inside the graph are the initial polymerization rate (in μmol s^−1^) for a specific photoinitiating system containing a photoinitiator and a co-initiator as indicated on the x and y axes.

Figure 1 shows the correlation between the initial rate of the TMPTA and Bis-GMA polymerization and the type of photoinitiator and co-initiator in the form of a heat map.

Photoinitiating systems containing two of the tested photoinitiators, DQ1 and DQ4, were used for the tests. DQ1 is a quinoline derivative, while DQ4 is based on the quinoxaline skeleton. In comparative tests, the efficiency of radical polymerization initiated by modified quinoline and quinoxaline photoinitiators was similar to the efficiency of the chain reaction obtained using commercial light absorber camphorquinone [42]. The data presented in Figure 1 clearly indicate that the initial polymerization rates of both TMPTA and Bis-GMA obtained for systems containing DQ1 and DQ4 are only slightly lower than those for CQ.

**Figure 2:**
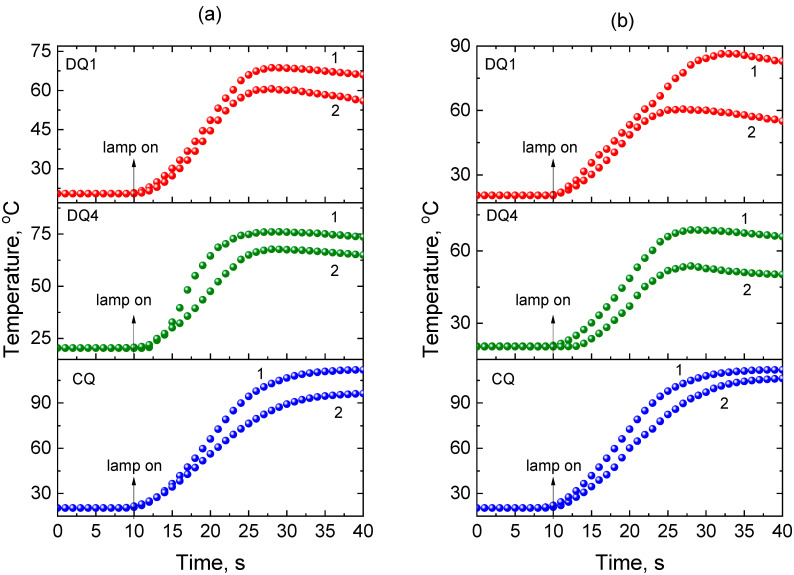
Kinetic curves recorded during the polymerization of (1) TMPTA and (2) Bis-GMA photoinitiated by quinoline [2,3-b]-1*H*-imidazo [1,2-a]pyridinium bromide (DQ1), 6-methyl-6*H*-indolo [2,3-b]quinoxaline (DQ4) and camphorquinone (CQ) in the presence of co-initiators: (**a**) (phenylthio)acetic acid (PhTAA) and (**b**) ethyl 4-dimethylaminobenzoate (EDMAB). The co-initiator concentration was 0.1 M, and the dental lamp light intensity was 20 mW/cm^2^.

Figure 2 shows the kinetic curves recorded during photoinitiation of free radical polymerization of TMPTA and Bis-GMA initiated by the synthesized DQ1 and DQ4 dyes as well as CQ in the presence of electron donors PhTAA and EDMAB.

The course of the kinetic curves presented in Figure 2 is similar for both monomers, i.e., Bis-GMA and TMPTA. In both cases, a hard polymer glaze is obtained. However, careful analysis of the data presented in Figure 1 and Figure 2 indicates that TMPTA hardens faster compared to commercial Bis-GMA. This is due to their chemical structure. Bis-GMA is an oligomer that has reactive double bonds at both chain ends, while TMPTA is a trifunctional acrylate ester monomer, which forms a polymer with a higher degree of cross-linking than Bis-GMA. In addition, TMPTA is useful for its low volatility. Generally, multifunctional acrylates reveal fast cure response because they polymerize by both chain growth and cross-linked polymerization. A very stiff, covalently cross-linked polymer network is then created [63]. This is very advantageous when using this monomer in in situ mass polymerization for dental fillings. Moreover, multifunctional acrylates polymerize faster than multifunctional methacrylates due to lower steric hindrance and higher radical stability [64,65]. The rapid bulk curing as well as the high hardness of the resulting polymer suggest that triacrylate polymer materials are suitable for in situ curing, which is essential for clinical applications in dentistry.

Furthermore, the potential clinical utility of TMPTA, as opposed to existing synthetic materials used in dentistry, was investigated by Mooney et al. [64] in terms of regenerative therapies. It has been proven that composite materials containing TMPTA can be a stem-cell-compatible restorative material used in direct contact with pulp tissues. This is indicated for treating pulp exposure injuries in permanent teeth because they cause irreversible damage that results in necrosis of the tissue and otherwise require root canal surgery [64,66]. TMPTA was also found to have antimicrobial properties as it is resistant to biofilm formation by Staphylococcus aureus and uropathogenic *Escherichia coli* [67]. It follows that triacrylate-based materials may be widely useful in dentistry, considering their many biological functions.

As shown in Figure 1 and Figure 2, (phenylthio)acetic acid (PhTAA) in the tested photocurable compositions is an effective co-initiator in combination with both the quinoline derivative (DQ1) and quinoxaline (DQ4) dyes. It has a wide range of applications, including in medicine. On this basis, new organotin derivatives were obtained that exhibit strong biological activity against cancer cell lines, especially chronic myeloid leukemia and lung cancer [68]. It is worth emphasizing that (phenylthio)acetic acid also has antibacterial activity. In combination with theophylline, it forms a cocrystal with increased activity against bacteria *Streptococcus pneumoniae* and *Pseudomonas aeruginosa* as well as mushrooms *Candida albicans* [69].

The research also indicates that the quinoline and quinoxaline derivatives (DQ1-DQ4) paired with PhTAA and EDMAB in a liquid organic matrix initiate the polymerization process at a similar rate to commercial camphorquinone (e.g., CQ-PhTAA 144.10 μmol/s vs. DQ4-PhTAA 115.30 μmol/s and DQ1-PhTAA 109.06 μmol/s). This means that the tested compounds are good photoinitiators of free radical polymerization of TMPTA operating in the visible light region.

To sum up, it can be stated that the triacrylate-based photocurable compositions we have designed are of immense importance as innovative restorative materials for conservative dentistry. They can be rapidly cured into bulk polymers, provide robust mechanical properties, and support regenerative functions. Furthermore, they can be placed directly at the dentin–pulp interface, suggesting that these materials could be further investigated for use in regenerative dentistry.

### 3.2. Weight Stability

The patient’s oral cavity is an extremely diverse environment, dominated primarily by aqueous solutions. Therefore, the properties of dental fillings may be influenced by many factors surrounding the restorative material. Through diffusion, they penetrate the structure of the polymeric materials, initiating chemical and physical processes. As a result, they lead to changes in their volume, plasticization, softening, swelling, hydrolysis, oxidation, etc. [36]. Research shows that polymeric restorative materials can absorb up to several percent of aqueous solutions. The saturation state is achieved after approximately 7–60 days of incubation [36,38,58,59,70]. The consequence of this phenomenon is swelling, which increases the dimensions of the material used [36], as well as its plasticization, causing a decrease in hardness [58]. Therefore, a crucial factor determining the durability of the filling used is the stability of the dental composite mass, most often determined by sorption and solubility.

#### 3.2.1. Sorption

The sorption of solvents contributes to the weakening of the mechanical strength of the composite due to the reduction in the stability of the connection of the filler particles with the organic matrix [40].

Tables S1 and S2 present the average values of sorption changes obtained for the tested samples after 7, 14, 28, 35, 42, 49, 56 and 63 days of storage in various solutions, i.e., in distilled water, 3% acetic acid solution, artificial saliva, 10% ethanol solution, heptane and in coffee, tea, red wine, and popular drinks such as Coca-Cola.

**Figure 3:**
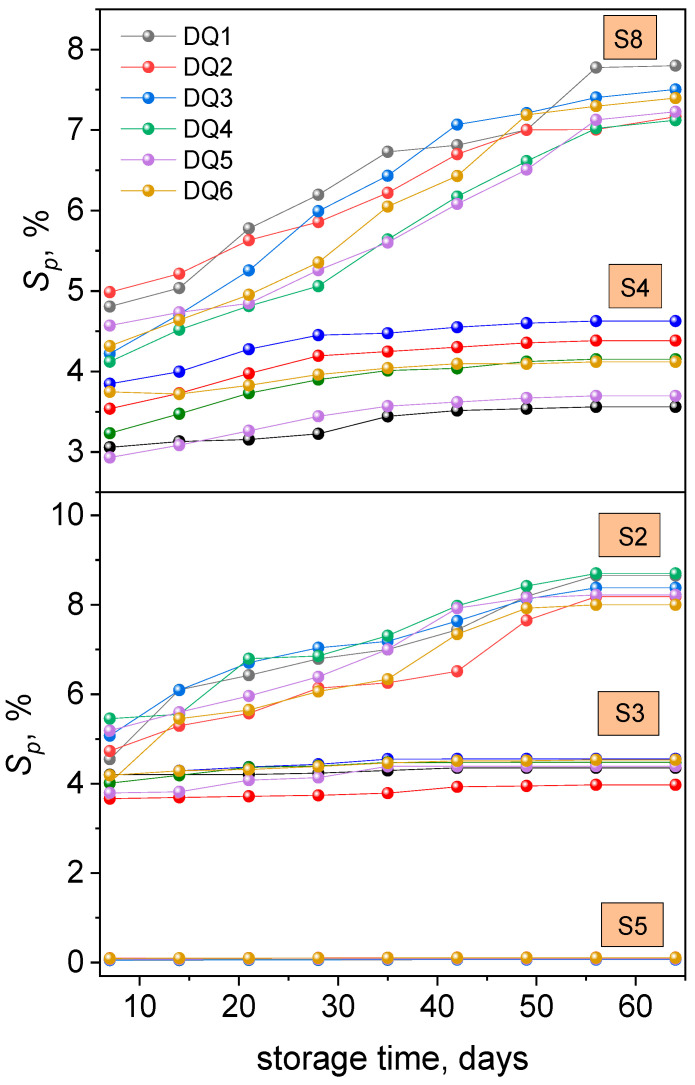
The course of the dependence of the average values of sorption changes in the tested materials on the conditioning time in a 3% acetic acid solution—S2, in artificial saliva—S3, in heptane—S5, in 10% ethanol solution—S4, and in Coca-Cola—S8.

The analysis of the data presented in Tables S1 and S2 allows to us state the lack of statistically significant differences in the sorption values of the tested samples after any soaking time under the influence of solutions such as distilled water, artificial saliva, tea, coffee, 10% ethanol solution, and red wine. This means that each of the tested samples, specifically DQ1-DQ5 and CQ, showed sorption values, the differences of which are not statistically significant. However, greater differences are observed in a clearly hydrophobic environment such as heptane (S5) and in the most aggressive solutions, i.e., 3% acetic acid (S2) and Coca-Cola (S8). The results are presented in the form of graphs of average values of sorption changes as a function of conditioning time for selected groups of solutions. The relationships presented in Figure 3 were determined in artificial saliva (as a representative of solutions such as distilled water (S1) and tea (S7), in which *S_p_* values did not show statistically significant differences), 3% acetic acid (S2), Coca-Cola (S8), 10% ethanol solution (S4) (as a representative of alcoholic beverages such as red wine (S9)), and heptane (S5).

The data presented in Tables S1 and S2 show that the average values of sorption changes (*S_p_*) increase with longer storage time of samples in solutions of 3% acetic acid—S2 and Coca-Cola—S8. In the case of samples stored in distilled water—S1, artificial saliva—S3, tea—S7, coffee—S6, and 10% ethanol solution—S4, after approximately 21 days of conditioning, sorption is established at a constant level. Longer storage of samples in these solutions does not affect the change in sorption. However, for samples conditioned in heptane—S5, this tendency is not observed; the sorption values are more or less stable with increasing storage time. By analyzing the curves presented in Figure 3 and the data contained in Tables S1 and S2, the sorption (*S_p_*) of the solution by the tested samples can be arranged in the following series: 3% acetic acid solution, Coca-Cola > distilled water, 10% ethanol solution, red wine > artificial saliva > tea, coffee > heptane. The values describing sorption for all solutions are positive.

The authors of other works determining the value of this parameter for commercial dental fillings most often used two fluids in the experiment: distilled water, and artificial saliva [36,40,58,60,71]. For commercial glass ionomer cements modified with resin, water sorption after 7 days of conditioning was in the range of 1 ÷ 9% [40]. In the case of composites based on Bis-GMA, water sorption is approximately 3% [58]. It was found that for a wide range of composite materials, this parameter can reach maximum values of up to 7% [36]. In our case, the sorption values of distilled water for the tested samples are in the range of 3.07 ÷ 4.80%, whereas for artificial saliva they are 3.66 ÷ 4.55%. This means that the tested material is comparable to commercial materials in terms of water and artificial saliva sorption.

#### 3.2.2. Solubility

The solubility of the material is caused by the presence of solutions in the vicinity of the filled cavity. It may lead to an unfavorable biological effect due to the washing out of unreacted substrates and polymer degradation products. It also leads to the destruction of its structure as a result of hydrolytic degradation [36,38].

**Figure 4:**
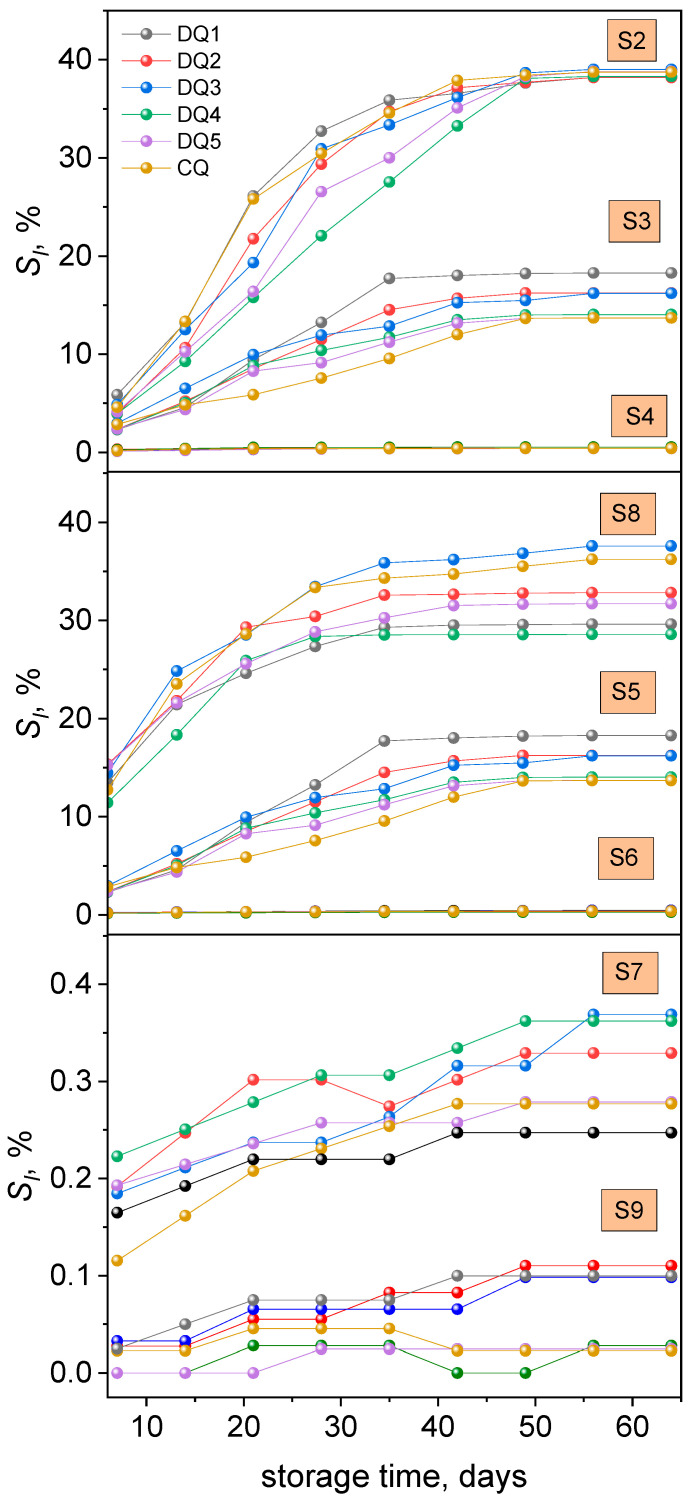
The course of the dependence of the average solubility values of the tested materials on the conditioning time in 3% acetic acid solution—S2, in artificial saliva—S3, in 10% ethanol solution S4, in Coca-Cola—S8, in heptane—S5, in coffee—S6, in red wine—S9, and in tea—S7.

Tables S1 and S2 present the average solubility values obtained for the tested samples in distilled water—S1, 3% acetic acid solution—S2, artificial saliva—S3, 10% ethanol solution—S4, heptane—S5 and in coffee—S6, tea—S7, Coca-Cola—S8 and red wine—S9, whereas Figure 4 show the correlation between the average solubility values of the tested samples and the time of conditioning in these solutions. Due to the lack of statistically significant differences, the course of the curves marked as “artificial saliva” should therefore be interpreted as characteristic also for distilled water.

Analyzing the data presented in Tables S1 and S2 and the correlation between the solubility change and the sample conditioning time (Figure 4), the solubility (*S_l_*) of the samples in various solutions can be set in the following series: 3% acetic acid > heptane > tea, coffee, distilled water, artificial saliva > 10% ethanol solution. The *S_l_* values are positive for all samples. The solubility of the tested samples in distilled water is in the range of 0.08 ÷ 1.62%, while in artificial saliva it ranges from 0.09% to 0.53%. The literature data indicate that the solubility of dental composites conditioned in aqueous solutions can reach a maximum value of 2%. In alcohol solutions and other organic solvents, the value of this parameter can be as high as 7% [36]. Lower solubility values of the tested samples than those reported in the literature indicate that our composite is much less soluble.

#### 3.2.3. Weight Change

**Figure 5:**
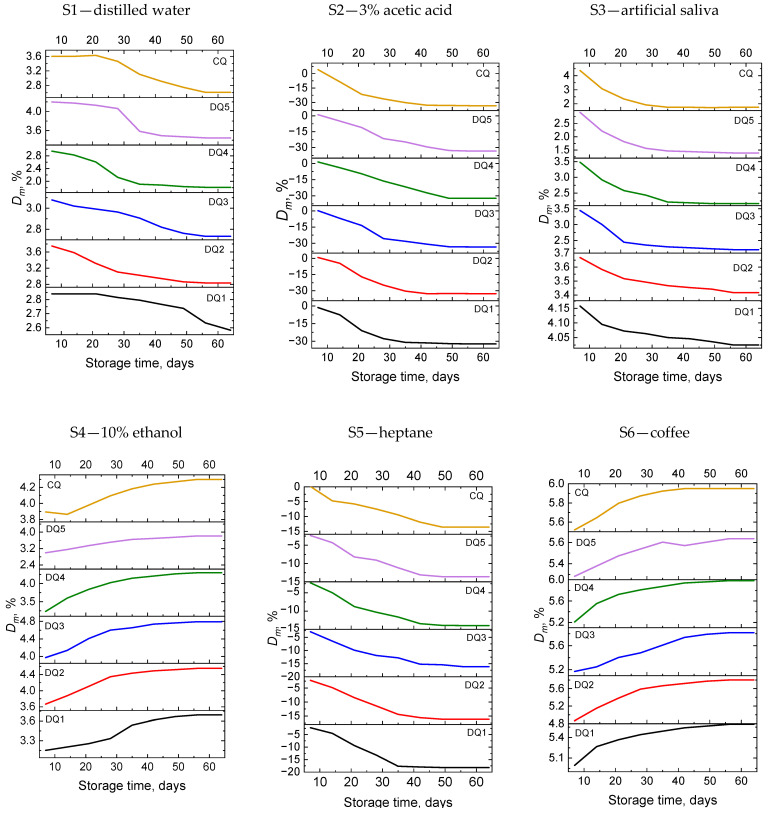

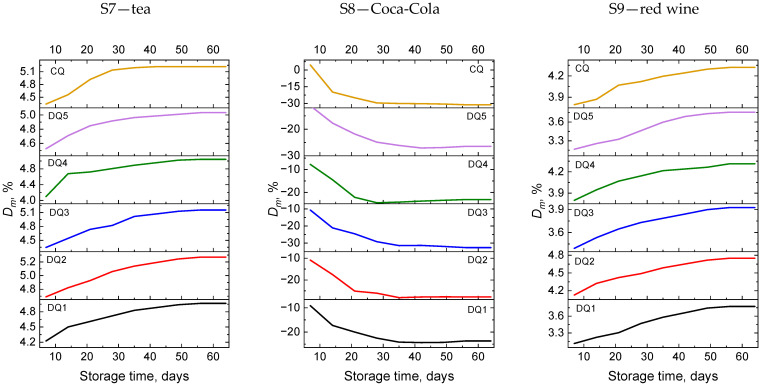
Correlation between the average values of the mass change in the tested materials and the conditioning time in the solutions simulating the oral cavity environment.

Tables S1 and S2 also present the average values of mass change obtained for the tested samples in distilled water—S1, 3% acetic acid solution—S2, artificial saliva—S3, 10% ethanol solution—S4, heptane—S5 and in coffee—S6, tea—S7, Coca-Cola—S8 and red wine—S9, while Figure 5 shows the relationship between the average values of mass change and the storage time of the samples in the solutions simulating the oral cavity environment.

The greatest mass changes occur in samples stored in tea, coffee, 10% ethanol solution, distilled water, and artificial saliva. The smallest changes occurred in a 3% acetic acid solution and the Coca-Cola, and minor changes occurred in heptane. The mass changes obtained for samples stored in a 3% acetic acid solution and Coca-Cola are negative.

The values of mass changes in distilled water for the tested material are in the range of 0.19 ÷ 3.74%, while for artificial saliva they are 0.17 ÷ 4.15%. In the case of commercial resin-modified glass ionomer cements often used as dental fillings, the values of this parameter are in the range of −4 ÷ 4 and −1 ÷ 7% for distilled water and artificial saliva, respectively [72]. The values obtained for our composite are comparable to the literature values.

## 4. Conclusions

In response to the constantly growing demand of the dental market for polymer composite materials for filling hard tooth tissues, research was undertaken to modify the composition of the liquid organic matrix in the photocurable composition. New photoinitiators based on a quinoline or quinoxaline skeleton (DQ1-DQ5) and a co-initiator—(phenylthio)acetic acid (PhTAA)—were used. Trimethylolpropane triacrylate (TMPTA) was applied as the monomer instead of Bis-GMA. Neutral IDG Dental glass functioned as an inorganic filler. These components created a photoinitiating composition that polymerized at a rate comparable to commercially available systems. Moreover, they seem to be safer for humans because they do not contain cytotoxic amines and Bis-GMA monomers, which often cause severe allergies. Therefore, newly designed composites based on TMPTA may be of immense importance as innovative restorative materials for conservative dentistry.

Moreover, durability tests for the developed composites based on the determination of mass stability, including sorption, solubility, and mass changes, provided results that are comparable to the literature values for other experimental and commercial dental fillings. The greatest stability of the tested composites was observed in heptane, which imitates fatty foods. In solutions such as distilled water (imitating hydrated food), artificial saliva, 10% ethanol (imitating food containing alcohol), red wine, tea, and coffee, the changes in sample properties were approximately similar, with the tested composites being the most susceptible to the effects of acidic foods and Coca-Cola.

In the next stages of research, we will address the topic of cytotoxicity and biochemical tests, the color stability of the obtained fillings, and their mechanical properties.

## Figures and Tables

**Table 1:** Structures of compounds for photoinitiated polymerization studies.

Photoinitiators	
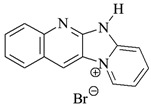 DQ1: quinoline [2,3-b]-1*H*-imidazo [1,2-a]pyridinium bromide	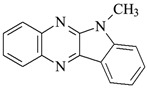 DQ4: 6-methyl-6*H*-indolo [2,3-b]quinoxaline
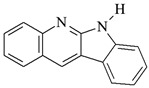 DQ2: 6*H*-indolo [2,3-b]quinoline	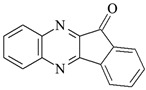 DQ5: 11*H*-indeno [1,2-b]qunioxalin-11-on
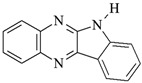 DQ3: 6*H*-indolo [2,3-b]quinoxaline	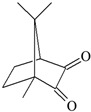 CQ: camphorquinone
Co-initiators	Solvent
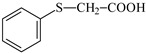 PhTAA: (phenylthio)acetic acid	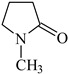 MP: 1-methyl-2-pyrolidinone
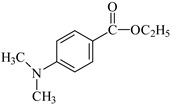 EDMAB: ethyl 4-dimethylaminobenzoate	
Monomers	
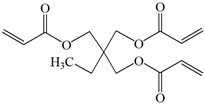 TMPTA: trimethylolpropane triacrylate	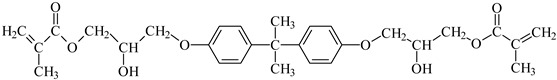 Bis-GMA: Bisphenol A glycerolate dimethacrylate

**Table 2:** Components of the developed and commercial compositions.

Developed Compositions						
No.	Organic Matrix (A), % *w/w*				Inorganic Filler (B), g	Organic Matrix to Filler Ratio (A:B), % *w/w*
	Photoinitiator	Co-Initiator	Monomer	Solvent		
1.	DQ1 0.05%	PhTAA 1.65%	TMPTA 88.20%	MP 10.10%	IDG 1.50	40:60
2.	DQ2 0.03%	PhTAA 1.65%	TMPTA 88.21%	MP 10.11%	IDG 1.50	40:60
3.	DQ3 0.03%	PhTAA 1.65%	TMPTA 88.21%	MP 10.11%	IDG 1.50	40:60
4.	DQ4 0.04%	PhTAA 1.65%	TMPTA 88.20%	MP 10.11%	IDG 1.50	40:60
5.	DQ5 0.04%	PhTAA 1.65%	TMPTA 88.20%	MP 10.11%	IDG 1.50	40:60
6.	CQ 9.91%	PhTAA 1.48%	TMPTA 79.50%	MP 9.11%	IDG 1.50	40:60
Commercial composition						
1.	CQ 9.88%	EDMAB 1.70%	Bis-GMA 79.40%	MP 9.02%	IDG 1.50	40:60

**Table 3:** Solutions used in mass stability tests of the tested materials.

Sample	Component	Simulated Environment
S1	distilled water	hydrated food with pH > 4.5
S2	3% acetic acid solution	hydrated food with pH < 4.5
S3	artificial saliva	saliva
S4	10% aqueous solution of ethyl alcohol	food containing alcohol
S5	heptane	fatty foods
S6	coffee	coffee
S7	tea	tea
S8	Coca-Cola	Coca-Cola drink
S9	red wine	wine

## Data Availability

The data is not publicly available apart from the data contained in the article or supplementary materials due to privacy.

## Supplementary Materials

The following supporting information can be downloaded at https://www.mdpi.com/article/10.3390/ma17236003/s1, Tables S1 and S2: Average values of solubility, sorption, and mass change of tested samples in various environments.
