# Supplementary material for: Modification of Light-Cured Composition for Permanent Dental Fillings; Mass Stability of New Composites Containing Quinoline and Quinoxaline Derivatives in Solutions Simulating the Oral Cavity Environment
Source: Materials (Basel). 2024 Dec 7;17(23):6003. doi: 10.3390/ma17236003 (PMC11643679; doi:10.3390/ma17236003)
Supplement: Supplementary file 1 [file materials-17-06003-s001.zip › materials-3341290-supplementary.pdf]

Supporting information

Modification of light-cured composition for permanent dental fillings; mass stability of new composites containing quinoline and quinoxaline derivatives in solutions simulating the oral cavity environment

Ilona Pyszka, Beata Jędrzejewska

Faculty of Chemical Technology and Engineering, Bydgoszcz University of Science and Technology, 85-326 Bydgoszcz, Poland;

*****Correspondence: Ilona.Pyszka@pbs.edu.pl (I.P.); Tel.: +48-52-374-9039 (I.P.), beata@pbs.edu.pl (B.J.) Tel.: +48-52-374-9046 (B.J.)

Table of contents **Page**

Table S1. Average values of solubility, sorption and mass change for the tested samples in distilled water, 3% acetic acid solution, artificial saliva, 10% ethanol solution and heptane S2

Table S2. Average values of solubility, sorption and mass change for the tested samples in coffee, tea, coca-cola and red wine S7

Table S1. Average values of solubility, sorption and mass change for the tested samples in distilled water, 3% acetic acid solution, artificial saliva, 10% ethanol solution and heptane

Parametr	Storage time, days	Solution				
		S1 (distilled water)	S2 (3% acetic acid)	S3 (artificial saliva)	S4 (10% ethanol)	S5 (heptane)
**DQ1**						
S_l, %	7	0.10	5.86	0.22	0.01	2.31
	14	0.44	13.21	0.28	0.02	4.57
	21	0.77	26.12	0.34	0.01	9.43
	28	0.85	32.74	0.34	0.01	13.24
	35	0.92	35.87	0.37	0.02	17.71
	42	0.98	36.58	0.41	0.02	18.02
	49	1.05	37.64	0.41	0.01	18.23
	56	1.08	38.20	0.44	0.01	18.28
	63	1.08	38.20	0.44	0.01	18.28
S_p, %	7	3.18	4.55	4.20	3.05	0.05
	14	3.19	6.10	4.20	3.13	0.05
	21	3.32	6.42	4.20	3.15	0.05
	28	3.35	6.79	4.23	3.22	0.06
	35	3.57	7.00	4.29	3.44	0.06
	42	3.69	7.44	4.35	3.51	0.06
	49	3.79	8.19	4.35	3.53	0.06
	56	3.79	8.65	4.35	3.56	0.06
	63	3.79	8.65	4.35	3.56	0.06
D_m, %	7	2.84	-1.37	4.15	3.15	-2.26
	14	2.84	-7.57	4.09	3.20	-4.52
	21	2.63	-21.04	4.03	3.25	-9.38
	28	2.58	-27.83	4.06	3.33	-13.18
	35	2.73	-31.04	4.09	3.53	-17.66
	42	2.8	-31.48	4.12	3.61	-17.96
	49	2.84	-32.07	4.12	3.66	-18.17
	56	2.81	-32.34	4.09	3.69	-18.22
	63	2.81	-32.34	4.09	3.69	-18.22

**DQ2**						
S_l, %	7	0.35	3.92	0.30	0.01	2.35
	14	0.54	10.65	0.38	0.01	5.20
	21	0.80	21.76	0.42	0.02	8.51
	28	1.18	29.37	0.40	0.02	11.50
	35	1.42	34.73	0.40	0.01	14.52
	42	1.58	37.15	0.42	0.01	15.68
	49	1.69	37.72	0.42	0.02	16.23
	56	1.79	38.20	0.42	0.02	16.23
	63	1.79	38.20	0.42	0.02	16.23
S_p, %	7	3.95	4.73	3.66	3.53	0.09
	14	3.98	5.29	3.69	3.72	0.09
	21	3.99	5.57	3.71	3.97	0.10
	28	4.15	6.14	3.74	4.19	0.10
	35	4.31	6.26	3.78	4.24	0.10
	42	4.39	6.51	3.92	4.30	0.10
	49	4.42	7.65	3.95	4.35	0.10
	56	4.50	8.18	3.97	4.38	0.10
	63	4.50	8.18	3.97	4.38	0.10
D_m, %	7	3.74	0.84	3.49	3.66	-2.25
	14	3.58	-4.57	3.44	3.87	-4.85
	21	3.31	-17.14	3.41	4.10	-8.42
	28	3.10	-24.75	3.46	4.34	-11.41
	35	3.02	-30.37	3.51	4.43	-14.43
	42	2.94	-32.77	3.62	4.49	-15.59
	49	2.86	-32.56	3.66	4.52	-16.14
	56	2.83	-32.69	3.69	4.55	-16.14
	63	2.83	-32.69	3.69	4.55	-16.14
**DQ3**						
S_l, %	7	0.08	4.86	0.20	0.02	2.95
	14	0.14	12.51	0.23	0.02	6.50
	21	0.17	19.32	0.29	0.05	9.95
	28	0.28	30.92	0.35	0.05	11.94
	35	0.37	33.37	0.38	0.02	12.84
	42	0.46	36.15	0.38	0.02	15.25

	49	0.52	38.67	0.41	0.05	15.47
	56	0.57	39.01	0.41	0.05	16.21
	63	0.57	39.01	0.41	0.05	16.21
S_p, %	7	3.07	5.07	4.18	3.84	0.05
	14	3.07	6.09	4.29	3.99	0.05
	21	3.07	6.70	4.37	4.27	0.06
	28	3.15	7.04	4.43	4.45	0.06
	35	3.19	7.18	4.55	4.47	0.06
	42	3.19	7.64	4.55	4.55	0.06
	49	3.19	8.13	4.55	4.60	0.06
	56	3.22	8.38	4.55	4.62	0.06
	63	3.22	8.38	4.55	4.62	0.06
D_m, %	7	3.07	0.22	3.44	3.97	-2.89
	14	3.02	-6.83	3.00	4.13	-6.45
	21	2.99	-13.52	2.44	4.40	-9.89
	28	2.96	-25.69	2.35	4.60	-11.89
	35	2.90	-28.20	2.29	4.65	-12.79
	42	2.82	-30.87	2.26	4.73	-15.19
	49	2.76	-33.24	2.23	4.76	-15.41
	56	2.73	-33.44	2.20	4.79	-16.15
	63	2.73	-33.44	2.20	4.79	-16.15
**DQ4**						
S_l, %	7	0.24	3.93	0.26	0.01	2.34
	14	0.50	9.26	0.38	0.01	5.04
	21	0.74	15.78	0.48	0.03	8.83
	28	1.39	22.07	0.51	0.03	10.39
	35	1.71	27.54	0.51	0.03	11.71
	42	1.85	33.26	0.53	0.01	13.50
	49	1.97	38.09	0.53	0.03	13.99
	56	2.02	38.31	0.53	0.03	14.03
	63	2.02	38.31	0.53	0.03	14.03
S_p, %	7	3.19	5.4	4.01	3.23	0.07
	14	3.24	5.55	4.18	3.47	0.07
	21	3.27	6.79	4.36	3.72	0.07
	28	3.45	6.85	4.39	3.89	0.07
	35	3.55	7.30	4.47	4.01	0.07

	42	3.67	7.97	4.47	4.03	0.08
	49	3.74	8.41	4.47	4.12	0.08
	56	3.77	8.70	4.47	4.15	0.08
	63	3.77	8.70	4.47	4.15	0.08
D_m, %	7	2.94	1.30	3.48	3.23	-2.27
	14	2.82	-3.93	2.92	3.59	-4.97
	21	2.60	-9.64	2.57	3.84	-8.76
	28	2.12	-16.33	2.43	4.02	-10.32
	35	1.90	-21.83	2.21	4.14	-11.64
	42	1.88	-27.47	2.19	4.20	-13.43
	49	1.83	-32.40	2.16	4.26	-13.92
	56	1.81	-32.43	2.16	4.30	-13.97
	63	1.81	-32.43	2.16	4.30	-13.97
**DQ5**						
S_l, %	7	0.13	4.12	0.09	0.02	2.39
	14	0.18	10.25	0.198	0.02	4.36
	21	0.31	16.38	0.29	0.02	8.27
	28	0.43	26.56	0.34	0.05	9.13
	35	0.99	30.01	0.37	0.05	11.22
	42	1.11	35.09	0.372	0.05	13.16
	49	1.38	38.28	0.397	0.05	13.66
	56	1.52	38.80	0.39	0.05	13.69
	63	1.52	38.80	0.39	0.05	13.69
S_p, %	7	4.16	5.19	3.79	2.93	0.07
	14	4.18	5.60	3.81	3.08	0.07
	21	4.27	5.96	4.07	3.26	0.07
	28	4.32	6.39	4.14	3.44	0.07
	35	4.42	7.00	4.39	3.56	0.08
	42	4.45	7.92	4.39	3.62	0.08
	49	4.69	8.15	4.39	3.67	0.08
	56	4.80	8.22	4.39	3.69	0.08
	63	4.80	8.22	4.39	3.69	0.08
D_m, %	7	4.19	1.12	2.90	2.99	-2.32
	14	4.17	-4.93	2.21	3.15	-4.29
	21	4.13	-11.09	1.81	3.34	-8.20
	28	4.06	-21.55	1.56	3.50	-9.06

	35	3.58	-24.73	1.46	3.64	-11.15
	42	3.49	-29.51	1.44	3.69	-13.09
	49	3.47	-32.80	1.41	3.75	-13.59
	56	3.44	-33.32	1.39	0.38	-13.62
	63	3.44	-33.32	1.39	0.38	-13.62
**CQ**						
	7	0.19	4.59	0.17	0.02	2.85
	14	0.27	13.33	0.31	0.01	4.82
	21	0.52	25.82	0.36	0.01	5.87
	28	0.76	30.45	0.39	0.02	7.56
S_l, %	35	1.12	34.58	0.39	0.02	9.56
	42	1.31	37.90	0.39	0.02	12.00
	49	1.42	38.42	0.41	0.01	13.64
	56	1.62	38.74	0.41	0.01	13.69
	63	1.62	38.74	0.41	0.01	13.69
	7	3.74	4.02	4.19	3.74	0.09
	14	3.74	5.45	4.29	3.72	0.09
	21	4.00	5.65	4.31	3.82	0.09
	28	4.09	6.06	4.38	3.96	0.10
S_p, %	35	4.10	6.33	4.46	4.04	0.10
	42	4.11	7.34	4.51	4.09	0.10
	49	4.06	7.92	4.51	4.09	0.10
	56	4.12	8.00	4.53	4.12	0.10
	63	4.12	8.00	4.53	4.12	0.10
	7	3.59	4.19	2.90	3.89	0.09
	14	3.59	-8.52	2.21	3.86	-4.73
	21	3.62	-21.54	1.81	3.98	-5.77
	28	3.46	-26.28	1.56	4. 09	-7. 47
D_m, %	35	3.10	-30.16	1.46	4.18	-9.47
	42	2.91	-32.98	1.44	4.23	-11.90
	49	2.74	-33.12	1.41	4.26	-13.55
	56	2.61	-33.42	1.39	4.29	-13.60
	63	2.61	-33.42	1.39	4.29	-13.60

Table S2. Average values of solubility, sorption and mass change for the tested samples in coffee, tea, coca-cola and red wine

Parametr	Storage time, days	Solution			
		S6 (coffee)	S7 (tea)	S8 (coca-cola)	S9 (red wine)
**DQ1**					
S_l, %	7	0.22	0.19	13.48	0.02
	14	0.27	0.24	21.43	0.02
	21	0.32	0.30	24.62	0.05
	28	0.37	0.30	27.34	0.05
	35	0.39	0.27	29.29	0.08
	42	0.42	0.30	29.52	0.08
	49	0.42	0.32	29.57	0.11
	56	0.44	0.32	29.62	0.11
	63	0.44	0.32	29.62	0.11
S_p, %	7	4.96	4.23	4.80	3.04
	14	5.26	4.54	5.03	3.14
	21	5.40	4.69	5.77	3.25
	28	5.51	4.79	6.19	3.40
	35	5.58	4.86	6.72	3.53
	42	5.65	4.94	6.81	3.61
	49	5.67	5.01	7.00	3.71
	56	5.72	5.04	7.7	3.74
	63	5.72	5.04	7.77	3.74
D_m, %	7	4.99	4.22	-9.11	3.11
	14	5.2	4.49	-17.27	3.22
	21	5.36	4.60	-20.00	3.30
	28	5.44	4.71	-22.55	3.46
	35	5.49	4.82	-24.19	3.57
	42	5.54	4.88	-24.36	3.66
	49	5.56	4.93	-24.26	3.74
	56	5.59	4.96	-23.68	3.77
	63	5.59	4.96	-23.68	3.77
**DQ2**					
S_l, %	7	0.20	0.18	4.98	0.03
	14	0.28	0.21	5.21	0.03

	21	0.3	0.23	5.63	0.06
	28	0.31	0.23	5.85	0.06
	35	0.33	0.26	6.21	0.06
	42	0.36	0.31	6.70	0.06
	49	0.36	0.31	7.00	0.09
	56	0.36	0.36	7.00	0.09
	63	0.36	0.36	7.00	0.09
S_p, %	7	4.83	4.65	4.22	3.99
	14	5.16	4.80	4.71	4.17
	21	5.40	4.92	5.25	4.29
	28	5.58	5.04	5.99	4.35
	35	5.68	5.13	6.43	4.44
	42	5.75	5.23	7.06	4.53
	49	5.80	5.28	7.20	4.59
	56	5.82	5.35	7.40	4.62
	63	5.82	5.35	7.40	4.62
D_m, %	7	4.86	4.69	-10.90	4.12
	14	5.15	4.82	-17.51	4.32
	21	5.38	4.92	-25.10	4.42
	28	5.59	5.05	-26.08	4.48
	35	5.66	5.13	-28.11	4.58
	42	5.72	5.19	-27.83	4.65
	49	5.77	5.24	-27.71	4.71
	56	5.79	5.27	-27.77	4.74
	63	5.79	5.27	-27.77	4.74
**DQ3**					
S_l, %	7	0.23	0.22	14.47	0.01
	14	0.28	0.25	24.84	0.01
	21	0.31	0.27	28.52	0.02
	28	0.31	0.30	33.43	0.02
	35	0.33	0.30	35.87	0.02
	42	0.36	0.33	36.20	0.01
	49	0.41	0.36	36.84	0.01
	56	0.43	0.36	37.5	0.02
	63	0.43	0.36	37.5	0.02
S_p, %	7	5.13	4.37	4.12	3.28

	14	5.25	4.58	4.52	3.41
	21	5.41	4.78	4.81	3.51
	28	5.48	4.89	5.06	3.62
	35	5.62	5.06	5.63	3.67
	42	5.76	5.14	6.17	3.72
	49	5.86	5.22	6.61	3.75
	56	5.91	5.24	7.02	3.80
	63	5.91	5.24	7.02	3.80
D_m, %	7	5.16	4.34	-10.70	3.39
	14	5.24	4.54	-21.12	3.53
	21	5.40	4.73	-24.56	3.64
	28	5.47	4.82	-29.19	3.72
	35	5.60	5.01	-31.46	3.78
	42	5.73	5.07	-31.35	3.84
	49	5.78	5.12	-31.93	3.89
	56	5.81	5.15	-32.60	3.92
	63	5.81	5.15	-32.60	3.92
**DQ4**					
S_l, %	7	0.15	0.19	11.42	0.01
	14	0.19	0.21	18.34	0.01
	21	0.21	0.23	25.91	0.01
	28	0.23	0.25	28.37	0.24
	35	0.25	0.25	28.37	0.24
	42	0.27	0.25	28.53	0.24
	49	0.27	0.27	28.56	0.24
	56	0.27	0.27	28.58	0.24
	63	0.27	0.27	28.58	0.24
S_p, %	7	5.36	4.29	4.12	3.79
	14	5.44	4.67	4.52	3.79
	21	5.61	4.73	4.81	3.90
	28	5.71	4.83	5.06	4.21
	35	5.79	4.90	5.63	4.28
	42	5.87	4.96	6.17	4.30
	49	5.88	5.04	6.61	4.32
	56	5.90	5.06	7.02	4.37
	63	5.90	5.06	7.02	4.37

D_m, %	7	5.20	4.09	-7.71	3.79
	14	5.55	4.67	-14.48	3.94
	21	5.72	4.72	-22.16	4.06
	28	5.81	4.80	-24.55	4.14
	35	5.87	4.89	-24.26	4.21
	42	5.93	4.95	-23.86	4.24
	49	5.96	5.02	-23.50	4.24
	56	5.98	5.04	-23.19	4.31
	63	5.98	5.04	-23.19	4.31
**DQ5**					
S_l, %	7	0.19	0.11	15.26	0.02
	14	0.25	0.16	21.60	0.2
	21	0.32	0.20	25.58	0.24
	28	0.35	0.23	28.83	0.04
	35	0.35	0.25	30.26	0.04
	42	0.35	0.27	31.52	0.02
	49	0.38	0.27	31.65	0.02
	56	0.38	0.27	31.71	0.02
	63	0.38	0.27	31.71	0.02
Sp, %	7	5.19	4.43	4.57	3.08
	14	5.34	4.65	4.73	3.17
	21	5.49	4.82	4.84	3.26
	28	5.58	4.90	5.25	3.38
	35	5.63	4.97	5.60	3.51
	42	5.60	5.01	6.08	3.57
	49	5.66	5.03	6.50	3.62
	56	5.69	5.05	7.12	3.64
	63	5.69	5.05	7.12	3.64
D_m, %	7	5.28	4.52	-11.20	3.16
	14	5.37	4.70	-17.71	3.25
	21	5. 47	4.84	-21.79	3.32
	28	5.53	4.91	-24.88	3.46
	35	5.60	4.96	-26.12	3.59
	42	5.56	4.98	-27.08	3.68
	49	5.60	5.00	-26.90	3.73
	56	5.63	5.03	-26.47	3.75

	63	5.63	5.03	-26.47	3.75
**CQ**					
S_l, %	7	0.20	0.16	12.73	0.02
	14	0.25	0.19	23.54	0.04
	21	0.30	0.21	28.61	0.07
	28	0.30	0.21	33.35	0.07
	35	0.35	0.21	34.31	0.07
	42	0.35	0.24	34.72	0.09
	49	0.37	0.24	35.51	0.09
	56	0.37	0.24	36.22	0.09
	63	0.37	0.24	36.22	0.09
S_p, %	7	5.23	4.16	4.31	3.65
	14	5.58	4.54	4.63	3.77
	21	5.76	4.89	4.95	3.98
	28	5.83	5.09	5.35	4.02
	35	5.92	5.17	6.04	4.09
	42	5.94	5.19	6.42	4.16
	49	5.97	5.22	7.18	4.21
	56	5.97	5.22	7.29	4.23
	63	5.97	5.22	7.29	4.23
D_m, %	7	5.51	4.34	4.50	3.79
	14	5.64	4.56	-19.82	3.87
	21	5.79	4.91	-24.89	4.06
	28	5.87	5.13	-29.57	4.11
	35	5.92	5.19	-30.08	4.19
	42	5.94	5.21	-30.24	4.24
	49	5.94	5.21	-30.52	4.29
	56	5.94	5.21	-31.21	4.31
	63	5.94	5.21	-31.21	4.31
